# Economic evaluations in community aged care: a systematic review

**DOI:** 10.1186/s12913-018-3785-3

**Published:** 2018-12-14

**Authors:** Norma B. Bulamu, Billingsley Kaambwa, Julie Ratcliffe

**Affiliations:** 1 Health Economics Unit, Flinders Health Care and Workforce Innovations, School of Medicine, Flinders University, Adelaide, SA Australia; 2 Institute for Choice, University of South Australia Business School, Adelaide, SA Australia

**Keywords:** Economic evaluation, Community aged care, Systematic review, Aged

## Abstract

**Background:**

This paper reports the methods and findings from a systematic review of economic evaluations conducted in the community aged care sector between 2000 and 2016.

**Methods:**

Online databases searched were PubMed, Medline, Scopus, and web of science, CINAHL and informit. Studies were included if they 1) were full economic evaluations that compared both the costs and outcomes of two or more interventions 2) in study population of people aged 65 years and over 3) dependent older people living in the community 4) alternatives being compared were care models or service delivery interventions in the community aged care sector (a group of programs that have been established as a support system to allow older people to remain living in their own homes for as long as possible, as an alternative to institutional or residential care) and 5) published in the English language between 2000 and November 2016.

**Results:**

Eleven studies reporting upon economic evaluations of service delivery interventions in community aged care were identified; the majority of which were undertaken in Europe. Critical appraisal of the identified studies highlighted the methodological rigour in these evaluations.

**Conclusion:**

This systematic review highlights the paucity of economic evaluation studies conducted to date in the community aged care sector. The findings highlight the importance of cost utility analysis methodology as it allows for a uniform outcome measure, that facilitates the comparison of different interventions. In addition, multi-attribute utility measures that represent those quality of life domains that are most important to older people should be used and attention must be paid to the inclusion of informal care costs and outcomes as this is a key resource in community aged care service delivery.

## Background

The World Health Organisation (WHO) has defined older people as persons who are 65 years or older [1] while the United Nations (UN) has classified persons over 60 years of age as older people [2]. By 2050, over 2 billion (22%) of the world’s population will be comprised of people aged over 60 years of age and of these, 402 million individuals will be over 80 years. Increased aging is associated with increases in dependency and frailty which creates additional demands upon health and aged care services [3]. Older age in Australia is defined as in the majority of developed countries as the retirement age or eligibility age for pension funds of 65+ years. Increased demand for aged care services coupled with scarce and constrained resources in this sector requires a review of existing policies and new approaches in the provision of aged care to ensure the efficient allocation of scarce resources i.e. in a manner that makes society better off rather than worse off [4, 5].

Aged care services in most developed countries are provided as government subsidies for older people to continue living at home, as community aged care services or in institutional care facilities or nursing homes as residential aged care services. Older people can continue living in the community with support for activities of daily living and or instrumental activities of daily living while those who are highly dependent and in need of intensive care to support their activities of daily living tend to live in institutions also referred to as residential aged care facilities or care homes or nursing homes [6–8]. Aged care services are provided under different jurisdiction internationally such as social care services in the UK [9], long-term care services in the USA [10] and most European countries [11]. Community aged care services are also referred to as home care services, home-based care, care at home or home and community-based services [12]. Policy reforms in community aged care service provision internationally led to the introduction of consumer directed models of care which aim to improve older people’s involvement in decision making with regard to the care that they receive [12–14]. However little evidence is currently available to assess the costs and outcomes associated with these policy reforms or to determine the cost effectiveness of models of care in the aged care sector [15, 16].

In contrast to the wide proliferation of economic evaluations reported upon in the health care sector, a paucity of economic evaluations have been conducted in community aged care [17]. Economic evaluation is defined as the comparative assessment of the cost and benefits of alternative interventions, also referred to as the comparators [18]. There are five main types of economic evaluation: cost minimisation analysis, cost effectiveness analysis, cost utility analysis, cost benefit analysis and cost consequence analysis [18]. The key feature that distinguishes the different types of economic evaluation is the unit for measuring the benefits of interventions in community aged care. Cost effectiveness analysis focuses upon a single measure of outcome, which is typically measured in natural units and is specific to the research question being addressed. Cost utility analysis is more generally focused on quantifying the quality of life benefits that older people may obtain from new innovations in aged care with the main measure of outcome being quality adjusted life years (QALYs) [18, 19].

The viewpoint or perspective of an economic evaluation is important as it determines the range of inputs/costs and outputs/outcomes to be considered in the economic evaluation. The societal perspective is the broadest perspective and is often advocated for use when evaluating publicly funded programs, where all costs and benefits to society as a whole irrespective of who incurs or receives them are considered [18, 20]. Costs are categorised according to where they are incurred e.g. community aged care, residential aged care, health care sector, client/family costs (the private costs incurred by the client and their family in receiving care, productivity losses as a result of the provision of informal care) [18].

The main objectives of this systematic review were to i) capture the available evidence relating to the application of economic evaluation in the community aged care sector and ii) provide a critical appraisal of previous full economic evaluation studies that compared both the costs and outcomes of two or more interventions for dependent older people in community aged care and published in the English language (where dependency was defined as needing some assistance to perform activities of daily living through the receipt of informal care and/or community aged care services) iii) examining to what extent informal care was included and the methods used to value informal care e.g. productivity losses. The review focussed only on full economic evaluations because these are preferred to partial evaluations by decision making bodies internationally including the Medical Services Advisory Committee (MSAC) in Australia and the National Institute for Health and Care Excellence (NICE) in the UK [21, 22].

## Methods

### Search strategy and selection criteria

**Database:** PubMed, Medline, Scopus, CINAHL, informit, and Web of Science

**Search terms:**Three key concepts were considered and incorporated

i. the population was defined by subject headings such as aged; aged, frail and keywords such as elder or old age or older person or people or adult
ii. economic evaluation methodology was defined by headings including economics; quality-adjusted life years; costs and cost analysis and keywords economic analysis or evaluation or model, cost effectiveness or cost utility or cost benefit, quality adjusted life years or QALY
iii. community aged care sector was defined by subject headings such as Homes for the Aged; Independent Living and keywords such as community care or home care or community aged care or home living or community living

The search strategy used in Medline is presented in Table 1 below.

**Table 1:** Medline search strategy

# ▲	Searches
1	(community care or home care or community aged care).tw.
2	((geriatric or elder or ‘older people’) adj2 (home* or apartment* or residence*)).tw.
3	(((home or community) adj5 (dwelling or based or setting*)) or (living adj5 (home or community or independent*))).tw.
4	((community or home* or respite or social or aged) adj5 (care* or welfare* or support*)).tw.
5	Homes for the Aged/ or Health Services for the Aged/ or Social Welfare/ or Community Health Services/ or Independent Living/
6	or/1–5
7	economics/ or Quality-adjusted life years/
8	exp “costs and cost analysis”/ or cost-benefit analysis/ or “cost of illness”/ or exp. health care costs/
9	“Value of Life”/ec [Economics]
10	((economic* adj1 (analys* or evaluat* or model*)) or (cost adj2 (effective* or utilit* or benefit or analysis or minimisation)) or (“quality adjusted life year*” or qaly)).tw.
11	or/7–10
12	Aged/
13	“aged, 80 and over”/ or frail elderly/
14	(elder* or geriatric* or old age* or ((old* or aged) adj (person or people* or adult*))).tw.
15	(aged adj (“65” or “70” or “75” or “80” or “85”)).tw.
16	or/12–15
17	6 and 11 and 16
18	limit 17 to (english language and yr = “2000 - Current”)

The study selection process followed the Preferred Reporting Items for Systematic Reviews and Meta-Analyses (PRISMA) guidelines for systematic reviews [23].

### Eligibility criteria

1) studies comparing both costs and outcomes of two or more interventions undertaken as stand-alone studies or alongside a clinical trial or other types of study design, 2) study population exclusive to people aged 65 years and over, 3) dependent older people living in the community, 4) the alternatives being compared (also referred to as comparators) were care models or service delivery interventions in the aged care sector, 5) published in the English language in peer reviewed journals between 2000 and November 2016.

Studies were excluded if: 1) both costs and outcomes were not considered and compared e.g. a cost analysis with no consideration of outcomes, effectiveness studies with no cost measurement, studies with no comparators, burden of disease or cost of illness studies 2) study population was not exclusive to people aged 65 years and over 3) study population was not based in the community 4) theory papers, letters, editorials, reviews, theses or dissertations and studies where full texts could not be obtained.

Two reviewers assessed the articles for eligibility with the second reviewer independently assessing 20% of the articles. Overall agreement between reviewers for this sub-sample was calculated using Cohen’s kappa statistic [24]. Both reviewers then undertook the quality assessment of included studies and one reviewer undertook the data extraction and data synthesis.

### Data extraction and synthesis

The data in included studies was synthesised narratively to identify the key methodological principles applied. Full text articles of included studies were read to obtain the following categories of information: study design and type of evaluation, key comparators, perspective/viewpoint of the study, the cost categories considered, type of costing used and the source of costing data, definition of outcomes and how they were measured, and the key results and conclusions of the study.

### Quality assessment

Economic evaluations that met the eligibility criteria were critically appraised using the critical appraisal checklist produced and disseminated by the University of Glasgow [25] that is based upon the guidelines developed by Drummond and colleagues [18].

Further assessment was undertaken to investigate the suitability of instruments applied in measuring quality of life and QALYs in the CUA studies and to examine the inclusion of and methods used to value informal care as a cost or outcome.

## Results

### Study selection process

**Fig. 1:**
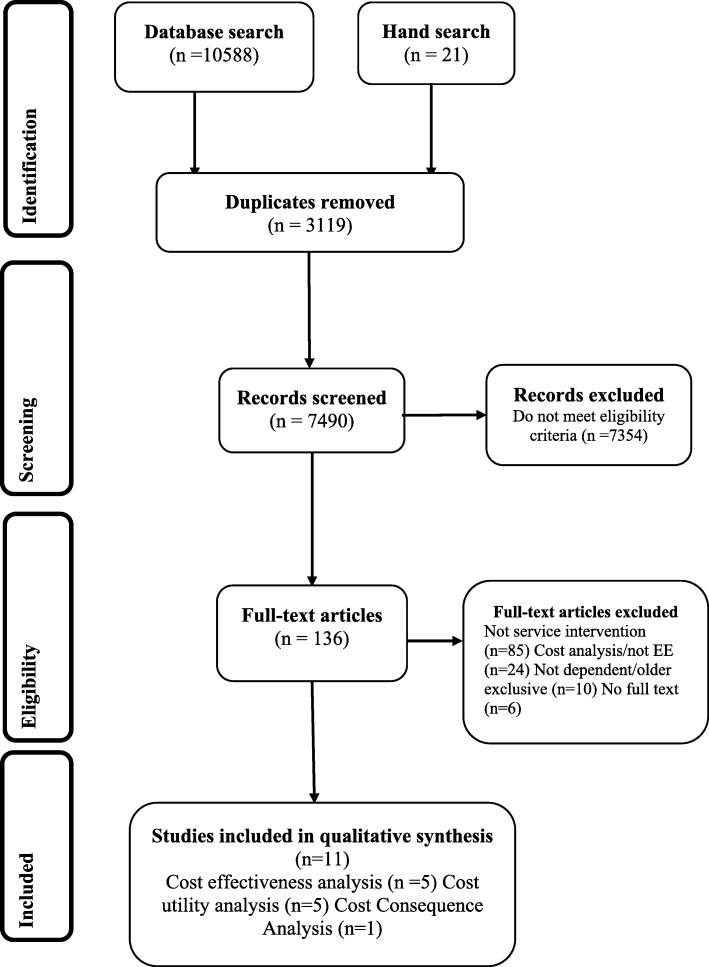
Study selection process

Study selection was divided into four main stages (see Fig. 1):

i. Identification: 10,588 papers were identified from the database search and an additional 21 from backward and forward searching the reference lists of the final studies accepted for the review, 3119 duplicate articles were removed.
ii. Screening: 7490 titles and abstracts were screened for eligibility; 7354 papers did not meet the eligibility criteria.
iii. Eligibility: 136 studies full texts articles were read and further assessed; 85 studies did not compare care models or service delivery interventions in community aged care, 24 were cost analysis studies while the population in 10 studies was not exclusive to older people and the full text could not be obtained in six studies.
iv. Included: 11 economic evaluation studies were included in the qualitative synthesis and narrative review. Five studies were CEAs, five CUAs and one CCA. The level of agreement relating to study exclusion and inclusion between the two reviewers was very high/almost perfect with a kappa statistic of 0.82 [26]

### Key findings

**Fig. 2:**
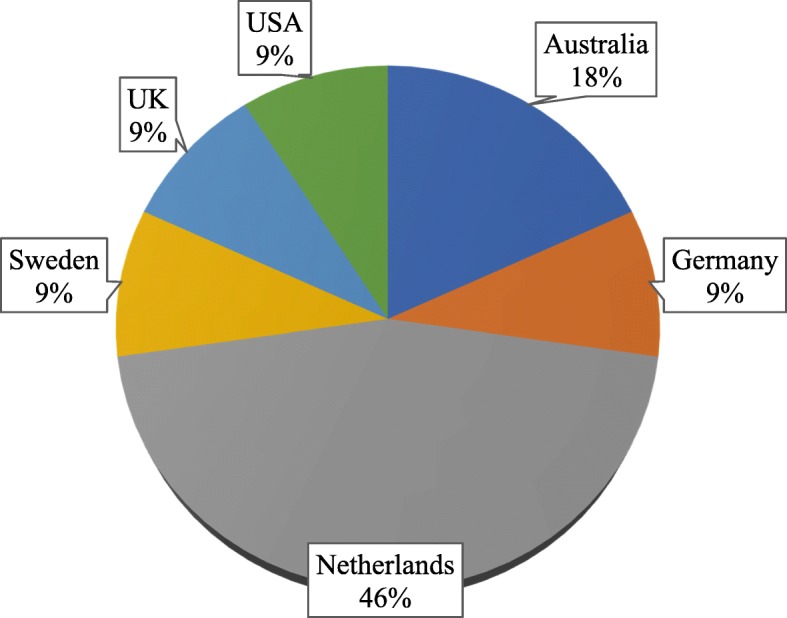
Geographical distribution of identified studies

The geographical distribution of the studies varied widely with nearly three quarters (8 studies) undertaken in Europe, refer to Fig. 2.

The majority (8 studies) of the economic evaluations were undertaken alongside randomised controlled trials (RCT). One economic evaluation was undertaken based on data from a retrospective cohort, and two were quasi-experimental studies. The sample sizes reported upon in the identified studies varied greatly from a minimum of 153 to a maximum of over 10,000 participants (see Table 2).

**Table 2:** Main characteristics of included studies

Title	Population Sample size and Country	Study design	Comparators	Perspective	Time horizon	Measure and Source of effectiveness data	Costs (Currency-Year)	Informal care-measurement and valuation	Source of cost data	Measure of Outcome	Conclusions
Cost utility analysis											
Cost-Utility Analysis of Preventive Home Visits program for Older Adults in Germany (Brettschneider et al., 2015)	80+ years *N* = 304 Germany	RCT	Preventive home visits vs usual care	S	18 months	Nursing home admissions RCT	Health care Client/family Informal care (Euro-2008)	Yes – patient recall, replacement cost method	Hospital, Nursing home and pharmacy Records, Self-report (resource use questionnaires)	QALY (EQ-5D-3 L)	Intervention unlikely to be cost effective
Cost utility analysis of case management for frail older people: effects of a randomised controlled trial (Sandberg et al., 2015)	65+ years *N* = 153 Sweden	RCT	Case management vs usual care	S	12 months	Healthcare utilisation RCT	Health care Other sectors, Client/family Informal care Intervention (Euro-2011)	Yes - patient recall, opportunity cost method	Hospital register, Community care records, Self-report	QALY (EQ-5D-3 L)	Intervention was cost neutral and did not seem to have affected health-related quality of life
Occupational therapy compared with social work assessment for older people. An economic evaluation alongside the CAMELOT randomised controlled trial (Flood et al., 2005)	65+ years *N* = 321 UK	RCT	Occupational therapist led vs social worker led assessment	PS	8 months	Dependency using the Community Dependency Index (CDI) RCT	Health care, Social care, Client/family (Pound sterling-2001)	No	Clinical records, Self-report (Cost questionnaire)	QALY (EQ-5D-3 L)	No difference in clinical and cost effectiveness
Cost-effectiveness of integrated care in frail elderly using the ICECAP-O and EQ-5D: does choice of instrument matter? (Makai et al., 2014a)	75+ years *N* = 352 Netherlands	Quasi-experiment	Integrated care vs usual care	S	3 months	ADL-functions, experienced health, mental well-being, social functioning, QES	Health care, Social care, Client/family, Intervention costs, Informal care (Euro-2011)	Yes – resource use questionnaire, NM	Patient health records, Self-report (care use questionnaire)	Capability (ICECAP-O) QALY (EQ-5D-3 L)	WICM maybe cost-effective based on capability QALYs
Cost effectiveness of the Walcheren Integrated Care Model intervention for community dwelling frail elderly (Looman et al., 2016)	75+ years *N* = 377 Netherlands	Quasi-experiment	Integrated care vs usual care	S	12 months	Functions, experienced health, mental well-being, social functioning QES	Health care, Social care, Client or family, Intervention costs, Informal care (Euro-2011)	Yes – resource use questionnaire, NM	Patient health records, Self-report (care use questionnaire)	QALY (EQ-5D-3 L)	The WICM is not cost-effective
Cost effectiveness analysis											
Effects on health care use and associated cost of a home visiting program for older people with poor health status: A randomized clinical trial in the Netherlands (Bouman et al., 2008)	70–84 years *N* = 330 Netherlands	RCT	Home visiting vs usual care	S	24 months	Health care use RCT	Health care, Intervention costs (Euro-2003)	No	Health use databases	Self-Rated Health (SRH)	Home visiting program did not appear to have any effect on the health care use of older people with poor health and had a low chance of being cost-effective
Cost effectiveness of a multi-disciplinary intervention model for community-dwelling frail older people (Melis et al., 2008)	70+ years *N* = 151 Netherlands	RCT	Multi-disciplinary intervention vs usual care	HS	6 months	Functional performance in ADL and IADL (GARS-3) and mental well-being (SF-20 MH scale) RCT	Health care, Social care (Euro-2005)	No	Primary care physician’s information system, Patient self-report	Successful treatment	Intervention is an effective addition to primary care for frail older people at a reasonable cost
Economic Evaluation of a Multifactorial, Interdisciplinary Intervention Versus Usual Care to Reduce Frailty in Frail Older People (Fairhall et al., 2015)	70+ years *N* = 241 Australia	RCT	Multi-factorial inter-disciplinary intervention vs Usual care for frailty	P S	12 months	Degree of frailty and disability RCT	Health care, Social care, Intervention costs (Australian dollar −2011)	No	Within trial service use database, Literature, Self-report	Transition out of frailty	A 12-month multifactorial intervention provided better value for money than usual care
Cost effectiveness of a chronic care model for frail older adults in primary care: economic evaluation alongside a stepped-wedge cluster randomised trial (van Leeuwen et al., 2015b)	65+ years *N* = 1147 Netherlands	RCT	Geriatric care model vs usual care	S	24 months	HRQoL (SF-12), and Functional limitations (Katz index) RCT	Health care, Social care, Intervention costs, Informal care (US dollar-2011)	Yes (52%) – Patient diary NM	Participant cost diaries Hospital and pharmacy registries	HRQoL (SF-12) QALY (EQ-5D-3 L) Functional limitations (Katz index)	Geriatric care model was not cost-effective compared to usual care after 24 months of follow-up
Cost effectiveness of a home-based intervention that helps functionally vulnerable older adults age in place at home (Jutkowitz et al., 2012)	70+ years 319 USA	RCT	Advancing Better Living for Elders (ABLE) vs Usual care	SP	2 years	Reduction in functional difficulty and mortality RCT	Intervention costs (US dollar-2010)	No	Within trial database, Literature	Life years saved	Investment in ABLE may be worthwhile depending on society’s willingness to pay
Cost consequence analysis											
Evidence for the long-term cost effectiveness of home care reablement programs	10,368 Australia		Post discharge reablement (PEP)**/**Community based reablement (HIP) vs Conventional home care service (HACC)	NM	5 years	Service provider	Home care service costs	No	WA Department of Health	Utilisation of home care services	Reablement services reduced the need for HACC services and this may contribute to containing the cost of aging

Study design: *RCT* = Randomised Control Trial, *QES* = Quasi experimental study, Perspective: *S*=Societal, *HS*=Health system, *PS*=Public sector (Health sector and social care sector), *SP*=Service provider (home care agency), *NM* = Not mentioned

The most prevalent types of evaluation were cost effectiveness analysis (5 studies) and cost utility analysis (5 studies) with one study reporting a cost consequence analysis. No studies were identified that used a cost- benefit approach. The interventions in 10 studies pertained to structures and processes of care/service delivery. Four studies assessed the value of preventative home visits compared to usual care where older people received care from their general practitioner (GP) or social services as and when it was needed [27–30]. Preventative home visits are designed to monitor the level of frailty and needs of older people, to preserve functional ability and subsequently delay admission to nursing homes or residential aged care facilities. One study assessed the cost effectiveness of re-ablement programs following hospital stay [31]. Four studies compared integrated multidisciplinary care interventions for frail older people to usual care where older people seek care from their GP as and when its needed. Integrated care programs were interventions that provided services to frail older people through coordinated multidisciplinary health teams [32, 33] and social care teams [34, 35]. Two studies compared interventions relating to the organisation of care; comparing different models for assessing older people’s eligibility for aged care services [36] and the value of case management in improving older people’s functional status and health care use [37]. Case management was an outreach service aimed at improving older people’s access to and continuity of health care services through needs assessment, care planning, care coordination as well as monitoring and evaluation of the older person’s needs.

The perspectives taken for the economic evaluations varied with six studies indicating that they had been undertaken from a societal perspective [27, 28, 32, 34, 35, 37], one study was undertaken from a health system perspective [30] and one study was undertaken from a service provider’s perspective [29]. Two studies considered the public sector (both health and social care) perspective [33, 36] whilst no particular perspective was indicated for the remaining study [31]. The time horizon for the economic evaluation was predominantly one year and under (6 studies).

A summary of the included studies is provided in Table 2.

### Application of economic evaluation methodology

#### Studies applying CUA methodology

As indicated previously, five studies applied a CUA methodology. One study evaluated the cost effectiveness of preventive home visits compared to usual care for community dwelling older people in Germany with time horizon of 18 months [28], two studies assessed the value of different processes of care; Sandberg et al. evaluated the cost utility of case management programs in Sweden over 12 months [37] while Flood and colleagues (2005) compared the cost effectiveness of occupational therapist (OT) led and social worker (SW) led assessment of the needs of older people in the UK over 8 months [36]. All three evaluations were each undertaken alongside RCTs. The final two studies applying CUA methodology analysed the cost effectiveness of the Walcheren integrated care model (WICM) alongside a quasi-experimental study among frail community dwelling older people in the Netherlands at two different time points; a follow up period of 3 months [35] and 12 months [34].

All studies applied a societal perspective with the consideration of client and family costs including informal care costs except in one study that applied a public sector perspective [36]. A micro-costing approach was used in all studies where client level data was obtained from records (hospital, nursing homes, social services and pharmacy) and self-report using resource use questionnaires.

The primary measure of outcome in all studies, the QALY, was measured using the EuroQol 5 Dimensions (EQ-5D-3 L) instrument, a multi-attribute utility measure of health status. One study [35] assessed quality of life using both the EQ-5D-3 L and the ICEpop CAPability measure for Older people (ICECAP-O), an older-person-specific measure of capability. Preventative visits did not improve quality of life and subsequently the intervention was not cost effective [28], and case management was found to be cost neutral compared to usual care [37] while OT led assessment was not cost effective compared to SW led assessment [36]. The last two studies highlighted the effect of the outcome measure on the results of an economic evaluation. There was no significant differences between WICM and usual care using the EQ-5D-3 L, a measure of health related quality of life (HRQoL) at 3 months [35] and even with a longer time horizon of 12 months [34]. However, there was a higher probability of cost effectiveness based on capability outcomes using the older-person-specific ICECAP-O [35].

#### Studies applying CEA methodology

Three studies applied CEA methodology. Two studies assessed the effectiveness of preventative home visiting programs to reduce frailty compared to usual care alongside RCTs in the Netherlands with time horizon of 24 months [27] and 6 months [30]. A societal perspective was adapted in the first study although only health care and social care costs were included with the exclusion of client/family costs as well as costs attributable to informal caring [27]. The second study assessed the Dutch Geriatric Intervention Program (DGIP), a preventative home visiting program, over 6 months under a health system perspective with the inclusion of health care and social care costs [30]. Both studies applied a micro-costing approach, using health records and client self-report for social care utilisation. Self-rated health (on ten-point Likert type scale ranging from poor to excellent) was the measure of outcome in the first study [27] while successful treatment was the measure of outcome in the second study; successful treatment was defined by improvement of functional performance in instrumental activities of daily living (assessed by the GARS-3) and mental wellbeing (assessed using the SF-20) [30]. The preventative program assessed by Bouman et al. was not found to be cost-effective compared to usual care while DGIP was cost-effective. Although these two studies evaluated closely similar interventions in the same setting (population and country), they cannot be directly compared because different measures of outcome were applied.

Another preventative intervention, Advancing Better Living for Elders (ABLE) was assessed alongside a RCT in the United States of America (USA) over a two-year study period [29]. The service provider’s perspective was taken in this study with only direct /intervention costs included using a micro-costing approach and life years saved (LYS) as a measure of outcome. Cost effectiveness was analysed under two scenarios: using within trial data and extrapolation to the real world setting where costs were elevated by 10%; ABLE was cost effective within the trial and in the real-world setting.

#### Studies applying CEA and CUA methodology

Two studies applied both CEA and CUA methodology in assessing the cost effectiveness of integrated care or multi-disciplinary approaches to the management of older people at home alongside RCTs. Fairhall et al. evaluated a multifactorial interdisciplinary health intervention for older people in Australia over 12 months [33] while van Leeuwen and colleagues assessed the cost effectiveness of the Geriatric Care Model (GCM) providing both health and social care services in the Netherlands over a 24-month period [32]. The public sector perspective was applied in Australia with the inclusion of health and social care costs. Both micro-costing and macro-costing approaches were used to obtain cost data with cost components including health and social care costs as well as direct intervention costs. The measure of effectiveness was transition out of frailty while quality of life was assessed using the EQ-5D-3 L. The intervention was found to be cost effective as more people transitioned out of frailty compared to usual care, however, there was no improvement in QALYs between the two groups and the ICER was not calculated for the CUA.

Van Leeuwen et al. applied a societal perspective including health and social care costs as well as the cost of informal care using a micro-costing approach (data obtained from hospital and pharmacy records as well as client cost diaries). Four measures of outcome were considered in this study; primary outcome was HRQoL measured by the short-form 12 health questionnaire (SF-12) and secondary outcomes were QALYs based on the EQ-5D-3 L and functional limitations in activities of daily living measured using the Katz basic activity of daily living scale. There were no differences in all outcomes between the two alternatives and the GCM was not found to be cost effective compared to usual care.

Similar to Makai et al. (2014) under CUA, there was no significant change in HRQoL between the intervention and control group using the EQ-5D-3 L [32, 33] and the SF-12 [32] in the two studies above, which may suggest that HRQoL measures may not be sensitive when applied in assessing interventions for older people.

#### Studies applying CCA methodology

To compare the value for money of a home care re-ablement program against conventional home and community care (HACC) in Australia, a CCA was conducted based on retrospective data over 5 years [31]. Re-ablement services were comprised of post-discharge re-ablement (PEP) for older people discharged from hospital back to the community and community based re-ablement (HIP) offered to community dwelling older people seeking to improve their levels of function. The service provider’s perspective was adapted for this study and only home care service costs were included, with the utilisation of HACC services as the measure of outcome. Re-ablement services reduced the need for HACC services and therefore contain the cost of aged care services.

### Critical appraisal of evaluations

Nine studies were satisfactorily conducted when assessed according to the critical appraisal criteria (all twelve questions). One study each were satisfactory in 11 questions [27] and 10 questions [31].

As the majority of economic evaluations were undertaken alongside clinical trials, the effectiveness of the intervention/s under consideration tended to be clearly established [27, 29, 30, 32, 33, 36–38]. The economic evaluations based on other study designs demonstrated effectiveness through a synthesis of evidence collated from review of literature [31, 34, 35]. Costs and outcomes were comprehensively identified and measured in all studies with the exception of one study that was reported as undertaken from a societal perspective but did not report costs associated with the provision of informal care [27]. All studies with time horizons beyond one year discounted both costs and outcomes at the appropriate rates based on the country where the study was undertaken. Notably, the CUA studies were all of high methodological quality ranking positive on all critical appraisal questions. A summary of the results of the critical appraisal assessment based on the University of Glasgow critical appraisal checklist is presented in Table 3.

**Table 3:** Results of the critical appraisal

Study reference	Is the EE likely to be usable			How were costs and outcomes assessed and compared						Will the results help in purchasing for local people		
	Q1	Q2	Q3	Q4	Q5	Q6	Q7	Q8	Q9	Q10	Q11	Q12
Cost-Utility Analysis Of Preventive Home Visits program for Older Adults in Germany [38]	Yes	Yes	Yes	Yes	Yes	Yes	No	Yes	Yes	Yes	Yes	Yes
Cost utility analysis of case management for frail older people: effects of a randomised controlled trial [37]	Yes	Yes	Yes	Yes	Yes	Yes	N/A^*^	No	Yes	Yes	Yes	Yes
Occupational therapy compared with social work assessment for older people. An economic evaluation alongside the CAMELOT randomised controlled trial [36]	Yes	Yes	Yes	Yes	Yes	Yes	N/A^*^	Yes	Yes	Yes	Yes	Yes
Cost-effectiveness of integrated care in frail elderly using the ICECAP-O and EQ-5D: does choice of instrument matter? [35]	Yes	Yes	Yes	Yes	Yes	Yes	N/A^*^	Yes	Yes	Yes	Yes	Yes
Cost effectiveness of the Walcheren Integrated Care Model intervention for community dwelling frail elderly [34]	Yes	Yes	Yes	Yes	Yes	Yes	N/A^*^	Yes	Yes	Yes	Yes	Yes
Effects on health care use and associated cost of a home visiting program for older people with poor health status: A randomized clinical trial in the Netherlands [27]	Yes	Yes	Yes	No	Yes	Yes	Yes	Yes	Yes	Yes	Yes	Yes
Cost effectiveness of a multi-disciplinary intervention model for community-dwelling frail older people [30]	Yes	Yes	Yes	Yes	Yes	Yes	N/A^*^	Yes	Yes	Yes	Yes	Yes
Economic Evaluation of a Multifactorial, Interdisciplinary Intervention Versus Usual Care to Reduce Frailty in Frail Older People [33]	Yes	Yes	Yes	Yes	Yes	Yes	N/A^*^	Yes	Yes	Yes	Yes	Yes
Cost effectiveness of a chronic care model for frail older adults in primary care: economic evaluation alongside a stepped-wedge cluster randomised trial [32]	Yes	Yes	Yes	Yes	Yes	Yes	Yes	Yes	Yes	Yes	Yes	Yes
Cost effectiveness of a home-based intervention that helps functionally vulnerable older adults age in place at home [29]	Yes	Yes	Yes	Yes	Yes	Yes	Yes	Yes	Yes	Yes	Yes	Yes
Evidence for the long-term cost effectiveness of home care re-ablement programs [31]	Yes	Yes	Yes	Yes	C	Yes	N/A^*^	N/A^*^	C	Yes	Yes	Yes

Q1: Well-defined question; Q2: Comprehensive description of alternative; Q3: Evidence of effectiveness; Q4: Important/ relevant outcomes and costs identified; Q5: Outcomes and costs measured accurately in appropriate units; Q6: Outcomes and costs valued credibly; Q7: Discounting; Q8: Incremental analysis of the outcomes and costs; Q9: Sensitivity analysis; Q10: Discussion of the results include issues that are of concern to purchasers; Q11: Conclusion justified by the evidence presented; Q12: Results applicable to local population; *N/A is considered as an answered question

#### Measuring QALYs and HRQoL

In measuring QALYs, all of the studies [28, 34–37] applied the EQ-5D measure. Two studies using HRQoL as a measure of effectiveness applied the SF-12 [32] and SF-20 [30]. The EQ-5D is a multi-attribute utility measure of quality of life assessing dimensions including mobility, self-care, usual activities, pain/discomfort and anxiety/depression [39]. The SF-12 and SF-20 are shorter versions of the generic HRQoL measure SF-36 which assesses eight dimensions of quality of life using 36 questions [40]. The SF-12 contains 12 questions assessing the original eight dimensions including physical functioning, role-physical, bodily pain, general health, vitality, social functioning, role-emotional and mental health [41] while the SF-20 has 20 questions assessing physical functioning, role functioning, bodily pain, current health perspective, social functioning and mental health [42].

#### Informal care

At least 30% of participants in all included studies reported themselves as having an informal carer but only 5 studies incorporated the cost of informal care. This could be attributed to the narrow perspectives applied in some studies, for example although 75% of participants in one study [30] reported themselves as having an informal carer, the cost of informal care was not considered because the study was conducted under a health sector perspective. Five of the six studies conducted under a societal perspective included the cost of informal care [28, 32, 34, 37, 43] but none considered the effect of the caring role on carer’s quality of life or informal care on the outcomes side of the equation. Informal care was measured through patient recall [28, 37] or resource use questionnaires completed by the client or the informal carer [32, 34, 43] and none reported whether informal care was provided at the same time as other activities such as leisure activities or joint production. It was valued using the opportunity cost method using the average wage rate [37] or the replacement cost method using the wage of a paid carer [28] but the method of valuation not mentioned in the remaining three studies. Informal care was a key cost driver in two studies; making the intervention costlier through increased use [32] and less costly through reduced use [37]. Sandberg et al., 2005 found no significant differences in costs or quality of life between the intervention and usual care but the need for informal care declined in the intervention group [37].

## Discussion

This systematic review has highlighted the paucity of economic evaluation studies conducted in the community aged care sector. The most prevalent types of economic evaluation methodologies applied were cost effectiveness analysis and cost utility analysis.

Whilst a study focusing upon CCA was included in this review, CCA does not aggregate costs and outcomes to give an indication of the incremental cost effectiveness associated with the competing alternatives [18, 44]. It has been suggested however, that this type of analysis may allow the decision maker more flexibility to select those components of costs and outcomes that may be relevant to their decision and compute a cost effectiveness ratio if desired [44].

As observed in some of the studies, measurement of outcomes in natural units is a limitation of CEA as there is no standard measure of outcome and this affects the comparability of results between studies [30, 33]. In contrast, CUA facilitates direct comparisons between competing interventions most often through the calculation of QALYs, a generic measure of outcome. Cost utility analysis is recommended as the preferred approach for the economic evaluation of interventions in aged care and has also been highlighted as a useful framework for the conduct of economic evaluations by key funding bodies internationally [45–48]. A key issue for the conduct of CUA in the community aged care sector is the identification of appropriate instruments that capture the breadth of quality of life as defined by older people [33, 35, 36, 49]. Whilst widely validated measures such as the EQ-5D were applied in the studies identified in this review, HRQoL measures primarily focus on health and physical functioning and do not specifically address dimensions of quality of life that are most important to older people and may not be sensitive to quality of life improvements in this population [19, 32, 35, 50]. Several commentators have argued that to comprehensively reflect quality of life benefits of interventions to older people, one requires an instrument that not only measures health and physical functioning but includes dimensions that are important to older people in receipt of aged care particularly psychosocial functioning, the ability to be independent, control over daily lives and involvement in decision making, and also defines health as a resource to help them achieve their goals and facilitate social and physical participation [50–53]. Two reviews of instruments suitable for use in economic evaluations involving older people have highlighted the potential benefits of the use of preference based instruments such as the ICECAP-O and the Adult Social Care Outcomes Toolkit (ASCOT) which both have a focus on wider quality of life attributes beyond health status [43, 54]. Depending on the intervention under review, such instruments may be applied in combination with HRQoL instruments [55–57].

Some commentators have also argued that the QALY metric may not be a suitable measure for older people as its calculation is based on a combination of quality of life and length of life, which by default discriminates against older people with a shorter life expectancy [52, 58, 59]. Yet the derivation of value sets for most instruments with the QALY scale has been predominantly based on populations with younger age groups (18–64 years) and less representation of older people (over 65 years) [5, 60].

The Second Panel on Cost-Effectiveness in Health and Medicine recommends that results of economic evaluations should be reported from a societal perspective as a reference case with the inclusion of informal care costs [61]. A review of the inclusion of informal care in applied economic evaluation found that only a small proportion of studies formally included informal care [62]. Our findings were consistent with this review. Although most study participants (at least 30% in all studies) reported themselves as having an informal carer, only five studies considered the cost of informal care and none formally considered the impacts upon carers. The time spent providing informal care (such as personal care and household tasks) is a resource that is used up as a result of caring, and so carers should arguably be considered as a cost in economic evaluation. The carer’s quality of life, however, may also be affected by a person’s condition, and so outcomes for carers may also be relevant in an economic evaluation. Within the CUA framework, a new instrument the Carer Experience Scale (CES) which has been specifically designed for carers to measure and value the impact upon caring associated the introduction of new interventions may be particularly helpful. However, it has been argued that incorporating both carer costs and outcomes in the cost effectiveness ratio (CER) may result in double counting as carers may have considered their quality of life when valuing their time [63]. Other commentators suggest that carer effects may be accounted for on the cost side of the equation if monetary methods of valuing benefits are used and on the effects side when non-monetary methods of valuing benefits are applied [64, 65]. Another possible option is to move beyond assessment of quality of life within CUA and incorporate the wider impacts of an intervention upon the caring role in monetary terms within the framework of cost benefit analysis [66].

Overall, most of the interventions included in this review were not found to be cost effective, which was largely attributed to the short time horizons applied in these studies. A longer time horizon is recommended for service delivery interventions that involve the integration of various sectors and building of networks in service delivery such as in the community aged care sector to permit the intervention to go beyond the teething problems and adjustment phases to then observe the benefits attributed to the intervention [34, 37, 67, 68].

Strengths of this review include the systematic approach to data collection using a comprehensive search strategy in multi-disciplinary health databases and the geographical variation in the studies included that allowed for a snapshot of the evaluation of aged care services internationally. However, it was also a challenge as the aged care system and definition of services varies from one country to another and affects the comparability of the different studies. The review was also limited to empirical research published in the English language (model-based studies and grey literature were excluded) and full-text that could be accessed through the university library system.

## Conclusion

In contrast to the high prevalence of economic evaluations conducted in the health care sector this systematic review has identified that relatively few economic evaluations have been conducted to date in the community aged care sector. The findings highlight the importance of cost utility analysis methodology as it allows for a uniform outcome measure, that facilitates the comparison of different interventions. Within cost utility analysis, multi-attribute utility measures that represent those quality of life domains that are most important to older people should be used and attention must be paid to the inclusion of informal care costs and outcomes as this is a key resource in community aged care service delivery. Future research should be directed towards developing methods and applications to facilitate the inclusion of carers effects in the economic evaluation of interventions for the community aged care sector.

## Abbreviations

ASCOT: Adult social care outcomes toolkit
CCA: Cost consequence analysis
CEA: Cost effectiveness analysis
CINAHL: Cumulative index to nursing and allied health literature
CUA: Cost utility analysis
EQ-5D-3: EuroQol 5 Dimensions 3 level
GP: General practitioner
HRQoL: Health related quality of life
ICECAP-O: ICEpop capability measure for older people
LYS: Life years saved
PRISMA: Preferred reporting items for systematic reviews and meta-analyses
QALY: Quality adjusted life years
RCT: Randomised controlled trial
U: United Kingdom
